# Clinical relevance of molecular testing methods in the diagnosis and guidance of therapy in patients with staphylococcal empyema: a systematic review and meta-analysis

**DOI:** 10.3389/fcimb.2022.758833

**Published:** 2022-07-29

**Authors:** Suvash Chandra Ojha, Ke Chen, Yue Yuan, Sarfraz Ahmed, Aijaz Ahmad Malik, Mehru Nisha, Yun-Jian Sheng, Changfeng Sun, Gang Wu, Cun-Liang Deng

**Affiliations:** ^1^ Department of Infectious Diseases, The Affiliated Hospital of Southwest Medical University, Luzhou, China; ^2^ Southwest Medical University, Jiangyang District, Luzhou, China; ^3^ Department of Basic Sciences, University of Veterinary and Animal Sciences Lahore, Narowal, Pakistan; ^4^ Center of Data Mining and Biomedical Informatics, Faculty of Medical Technology, Mahidol University, Bangkok, Thailand; ^5^ Investigative Biomedical Science Research Cluster, Institute of Medical Science Technology, Universiti Kuala Lumpur, Kajang, Selangor, Malaysia

**Keywords:** pleural infection, NAAT accuracy, anti-staphylococcal therapy, systematic review, meta-analysis

## Abstract

**Background:**

Efficient detection tools for determining staphylococcal pleural infection are critical for its eradication. The objective of this meta-analysis was to assess the diagnostic utility of nucleic acid amplification tests (NAAT) in suspected empyema cases to identify staphylococcal strains and avoid unnecessary empiric methicillin-resistant *Staphylococcus aureus* (MRSA) therapy.

**Methods:**

From inception to July 24, 2021, relevant records were retrieved from PubMed, Embase, Scopus, Web of Science, and the Cochrane Library. The quality of studies was determined using the QUADAS-2 tool. The pooled sensitivity, specificity, positive likelihood ratio (PLR), negative likelihood ratio (NLR), diagnostic odds ratio (DOR), and hierarchical summary receiver operating characteristic (HSROC) curve for NAAT’s diagnostic performance were evaluated using an HSROC model.

**Results:**

Eight studies comprising 424 samples evaluated NAAT accuracy for *Staphylococcus aureus* (SA) identification, while four studies comprising 317 samples evaluated methicillin-resistant *Staphylococcus aureus* (MRSA) identification. The pooled NAAT summary estimates for detection of both SA (sensitivity: 0.35 (95% CI 0.19–0.55), specificity: 0.95 (95% CI 0.92–0.97), PLR: 7.92 (95% CI 4.98–12.59), NLR: 0.44 (95% CI 0.14–1.46), and DOR: 24.0 (95% CI 6.59–87.61) ) and MRSA (sensitivity: 0.45 (95% CI 0.15–0.78), specificity: 0.93 (95% CI 0.89–0.95), PLR: 10.06 (95% CI 1.49–67.69), NLR: 0.69 (95% CI 0.41–1.15), and DOR: 27.18 (95% CI 2.97–248.6) ) were comparable. The *I* ^2^ statistical scores for MRSA and SA identification sensitivity were 13.7% and 74.9%, respectively, indicating mild to substantial heterogeneity. PCR was frequently used among NAA tests, and its diagnostic accuracy coincided well with the overall summary estimates. A meta-regression and subgroup analysis of country, setting, study design, patient selection, and sample condition could not explain the heterogeneity (meta-regression *P* = 0.66, *P* = 0.46, *P* = 0.98, *P* = 0.68, and *P* = 0.79, respectively) in diagnostic effectiveness.

**Conclusions:**

Our study suggested that the diagnostic accuracy of NAA tests is currently inadequate to substitute culture as a principal screening test. NAAT could be used in conjunction with microbiological culture due to the advantage of faster results and in situations where culture tests are not doable.

## Introduction

Pleural empyema is a serious complication of bacterial pneumonia. Staphylococcal strains, particularly methicillin-resistant *Staphylococcus aureus* (MRSA), are a leading cause of morbidity and mortality in sub-tropical areas (Asai et al., 2017; Chen et al., 2021). In recent years, pathogen identification has primarily relied on direct Gram stain and routine microbiological culture to determine the etiology of the empyema; however, a microbiological diagnosis cannot be made in up to 40% of cases of pleural infection using standard pleural fluid culture techniques (Hassan et al., 2019; Kanellakis et al., 2022). Poor detection rates are most likely due to a combination of prior antimicrobial therapy before obtaining pleural fluid samples for culture, low microbial concentration in pleural effusion, and possibly causal agents that are difficult to isolate in the laboratory due to stringent requirements (Insa et al., 2012; Hassan et al., 2021). Additionally, the traditional culture-based approach, which includes growth-based assays, colony morphology, and microdilution resistance tests, can be laborious and time-consuming. Even with a positive microbiological culture, it takes 48-72 hours for staphylococcal culture and antibiotic susceptibility testing to identify the causative organism. The high rate of culture-negative cases complicates clinical care and antibiotic selection, causing patients to miss out on the best chance of treatment. Therefore, clinical suspicion of staphylococcal infection is critical for facilitating diagnostic and therapeutic intervention in patients with pleural infection risk factors.

In patients with staphylococcal empyema, prompt drainage of infected fluid and timely initiation of anti-staphylococcal treatment are critical components of infection management (Hassan et al., 2021). However, selecting empirical anti-staphylococcal therapy is tricky because, in addition to staphylococcal strains, pleural empyema is caused by a variety of pyogenic bacteria, including *S. pyogenes*, *S. pneumoniae*, *H. influenzae*, anaerobes, and others (Hassan et al., 2019). Vancomycin or linezolid have been used as the primary parenteral therapy for patients with suspected staphylococcal empyema (Liu et al., 2011; Zhang and Zhang, 2021), with the understanding that postponements in initiating effective anti-staphylococcal agents may influence patient outcomes. Once microorganism identification and susceptibility are established, treatment can be tailored to target isolated bacteria, including vancomycin cessation when methicillin-sensitive *Staphylococcus aureus* (MSSA) is present. While this method is safe, it exposes the patient to broad-spectrum antibiotic overuse. Even short anti-MRSA treatment courses can alter host flora, expose patients to drug-induced toxicity, increase multidrug-resistant pathogens, cause treatment-related side effects, and elevate hospitalization costs (Zhang et al., 2015). When it comes to treating MSSA infections, oxacillin is more effective than the commonly prescribed antibiotic vancomycin (Stryjewski et al., 2007). If the initial antibiotics are grossly inadequate and are modified after the diagnostic tests are available, the mortality rate does not significantly improve. Therefore, striking a balance between these two competing interests, namely the need for comprehensive coverage while avoiding unnecessary medications, is becoming increasingly important.

Molecular diagnostic tools, which typically have a quicker response time, may aid in the establishment of an etiological diagnosis to help guide patient management. Several studies have shown that nucleic acid amplification tests (NAAT) can identify and guide treatment for staphylococcal infections in fluids like bronchoalveolar lavage (BAL), tracheal aspirate (TA), sputum, and blood (Chen et al., 2021; Chen et al., 2021). However, to the best of our knowledge, there is no published information on how well the NAAT assay works for finding pathogens in pleural fluids. In contrast to traditional culture-based methods, NAAT detects bacterial deoxyribonucleic acid (DNA) rather than viable bacteria, and thus is less influenced by pre-administration of broad-spectrum antimicrobials. Furthermore, NAAT detection of the *mecA* gene is widely regarded as the gold standard for MRSA diagnosis, which is critical for guiding therapy and avoiding unnecessary patient treatment. Many studies have evaluated the relevance of molecular techniques for empyema assessment, including conventional polymerase chain reaction (PCR) (Menezes-Martins et al., 2005; Utine et al., 2008; Feris-Iglesias et al., 2014; Amin et al., 2019), nested PCR (Blaschke et al., 2011), Unyvero multiplex PCR (Papan et al., 2018), real-time PCR (Tchatchouang et al., 2019), Septi*F*ast (Sancho-Tello et al., 2011), and 16S ribosomal ribonucleic acid (rRNA) metagenomic analysis (Dyrhovden et al., 2019), but research on the importance of these tests to timely staphylococcal empyema management is sparsely distributed. Given the importance of clinical decision-making in patients with staphylococcal empyema, we performed a meta-analysis to compare the diagnostic accuracy of NAAT to microbiological culture for *Staphylococcus aureus* (SA) and MRSA detection.

## Methods

### Search strategy

The study followed the Preferred Reporting Items for Systematic Reviews and Meta-Analyses (PRISMA) criteria for diagnostic test accuracy (McInnes et al., 2018). A computer-assisted literature search was conducted using PubMed, Embase, Scopus, Web of Science, and the Cochrane Library to identify relevant studies published between the establishment of the library and July 24, 2021. The following strategy was used in conducting the literature search: (‘*Staphylococcus aureus’* OR ‘*S. aureus*’ OR ‘Methicillin-resistant *Staphylococcus aureus*’ OR ‘MRSA’ OR ‘Staphylococcal empyema’) AND (‘Empyema’ OR ‘Pleural effusion’ OR ‘Empyema thoracis’ OR ‘Parapneumonic effusion’ OR ‘PPE’ OR ‘Pleural infection’ OR ‘Pleuritis’ OR ‘Pleurisy’ OR ‘Pleural fluid’) AND (‘Nucleic acid amplification’ OR ‘NAAT’ OR ‘Molecular assay’ OR ‘Loop-mediated isothermal amplification’ OR ‘LAMP’ OR ‘Polymerase chain reaction’ OR ‘PCR’ OR ‘Ligase chain reaction’ OR ‘LCR’ OR ‘Real-time PCR’ OR ‘qPCR’ OR ‘RT-PCR’ OR ‘Xpert’ OR ‘GeneXpert’ OR ‘Amplicor’ OR ‘SeptiFast’ OR ‘ProbeTec’ OR ‘Roche’ OR ‘Gen-Probe’ OR ‘FilmArray’ OR ‘Cepheid’ OR ‘Abbott’ OR ‘hyplex StaphyloResist’ OR ‘GeneOhm’ OR ‘LightCycler’) AND (‘Sensitivity’ OR ‘Specificity’ OR ‘Accuracy’). Additionally, we manually searched the reference lists of all the studies shown in **Supplementary table 1** and relevant reviews to identify potentially eligible studies.

### Study selection

The search terms were used to conduct a comprehensive search of electronic databases for all relevant citations, and duplicates were manually removed using the EndNote X9 software (Thomson Reuters, New York, NY, USA). The records were initially screened by looking at their titles and abstracts, and irrelevant studies were excluded from further analysis. The full text of presumably eligible studies was retrieved and meticulously analyzed for accuracy. All studies that met the standard empyema definition, including fever, chest pain, coughing, and dyspnea, were included. The evidence gathered by two separate researchers (S.C.O. and K.C.) was compared, and any discrepancies in the comparisons were resolved through mutual agreement.

### Inclusion criteria

Inclusion criteria were as follows: (i) assessment of the diagnostic accuracy of NAAT for diagnosing staphylococcal empyema; (ii) individuals suspected of having pleural empyema and Parapneumonic effusion (PPE); (iii) NAAT accuracy in the pleural fluid as the index test; (iv) use of microbiological culture as the reference standard for identifying SA and MRSA; (v) inclusion of sensitivity, specificity, or sufficient information to construct 2×2 contingency tables.

### Exclusion criteria

Reviews, meta-analyses, letters to the editor, conference proceedings, case reports, editorials, animal experiments, mechanism studies, and non-English publications were excluded from the study. Studies that did not provide information on sensitivity and specificity, such as mutation detection, mechanism, and comparison of different NAA tests, were excluded from consideration. Non-interpretable test results were excluded, as were studies that did not provide information on the diagnostic accuracy of NAAT in detecting staphylococcal strains in suspected patients using both the index test and the microbiological reference standard.

### Data extraction

Two investigators (S.C.O. and K.C.) independently extracted data from selected articles. Dispute resolution was facilitated through open discussion and consensus. Author, publication year, country, setting, study type, sample condition, sample size, NAAT specifics, potential features, sensitivity, specificity, true-positive, false-positive, false-negative, and true-negative rates for diagnosing staphylococcal empyema were extracted from eligible studies. Based on available data from qualifying studies, contingency tables for NAAT performance compared to microbiological reference standards were constructed. Studies that included both SA and MRSA datasets in the same study were treated as separate studies.

### Quality assessment

The methodological quality of the studies was determined using the Quality Assessment of Diagnostic Accuracy Studies-2 (QUADAS-2) method (Whiting et al., 2011). The quality assessment was conducted independently by two reviewers. The risk of bias was assessed in four QUADAS-2 domains, including patient selection, index test, reference standard, and flow and timing. Applicability concerns were investigated in three QUADAS-2 domains, including patient selection, index test, and reference standard. The spectrum and selection biases of participants were determined. Each domain was evaluated for bias risk using signalling questions that can be answered with “yes,” “no,” or “unclear” and are categorized as “low,” “high,” or “unclear,” respectively. A third reviewer (S.A.) was consulted in the event of an unresolved disagreement.

### Statistical analysis

Statistical analyses were performed using RevMan (version 5.4; Nordic Cochrane Centre, Copenhagen, Denmark), Meta-DiSc (version 1.4; Cochrane Colloquium, Barcelona, Spain), and STATA (version 16 SE; Stata Corporation, College Station, TX, USA). The values of true positives (TP), false negatives (FN), false positives (FP), and true negatives (TN) were retrieved from each of the included studies. RevMan was used to assess the methodological quality of included studies and generate forest plots to display summary estimates (Cochrane, 2008). The random-effect model was used in Meta-DiSc 1.4 (Cochrane Colloquium, Barcelona, Spain) to generate pooled summary estimates of specificity, sensitivity, likelihood ratios (LR), diagnostic odds ratio (DOR), and data heterogeneity (Zamora et al., 2006). The use of LR was rationalized because, unlike predictive values, it is unaffected by disease prevalence (Sedighi, 2013). Since the area under the hierarchical summary receiver operating characteristic (HSROC) curve is a global indicator of overall effectiveness, the HSROC curve was used to assess the assay’s impact, with an area under the curve (AUC) of 1 indicating superior discriminatory capabilities (Moses et al., 1993). The heterogeneity among the studies was evaluated using the *I*-square (*I* ^2^) statistics, where *I* ^2^ values <40% indicate low heterogeneity, <60% indicate moderate heterogeneity, <90% indicate substantial heterogeneity, and values >90% indicate considerable heterogeneity (Higgins et al., 2019). Additionally, we anticipated heterogeneity in categorical covariates of included studies. Therefore, we defined subgroups based on country, setting, study design, patient selection, and sample condition, assuming that the pooled sensitivity and specificity varied by subgroup. A meta-analysis for predefined subgroups was only carried out if at least three studies were available (Zamora et al., 2006). We used a bivariate random-effects model, an integrated approach in Meta-Disc, and conducted meta-analyses using the meta-regression option. Furthermore, Deek’s funnel plot asymmetry test was used to assess publication bias (Deeks et al., 2005). The STATA software with the *midas* package was used to assess publication bias. A two-sided *P*-value of <0.05 was generally regarded as statistically significant.

## Results

### Literature selection

**Figure 1:**
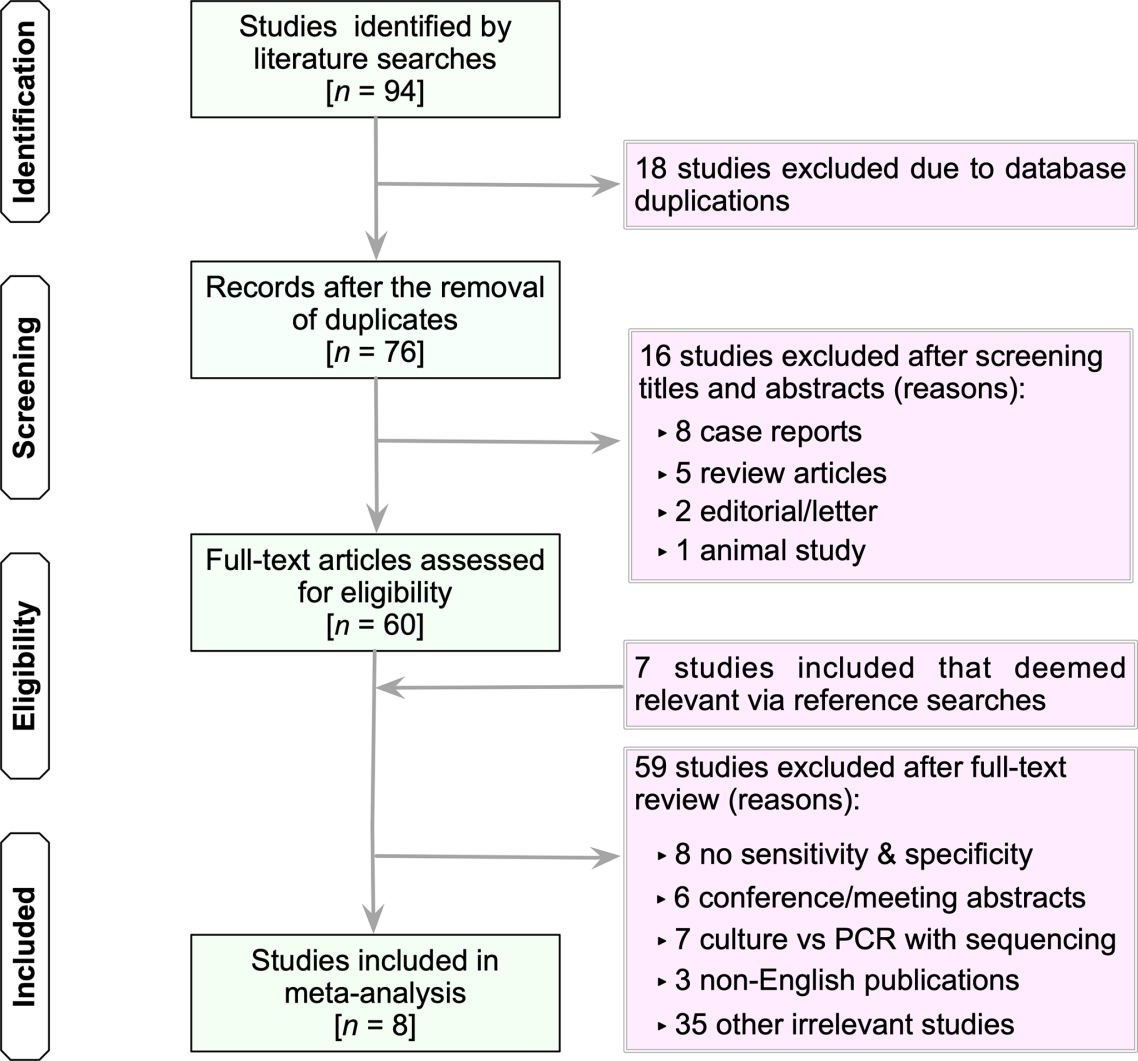
Flow chart of study selection. The term “irrelevant studies” in the figure refers to studies that did not provide sufficient information to construct 2×2 contingency tables. n, number; PCR, polymerase chain reaction.

Database searches yielded 94 studies (PubMed, 28; Embase, 10; Scopus, 48; Web of Science, 6; and the Cochrane Library, 2) (**Figure 1**). The first step was to remove 18 duplicate articles manually. Subsequently, 76 studies deemed potentially relevant were subjected to a full-text review based on their titles and abstracts. From reference searches, seven articles were chosen based on their relevance. **Supplementary Table S1** summarizes the reviewed studies and the reasons why these studies were excluded (*see* **Supplementary Table S1**). Finally, eight publications fulfilled the inclusion criteria and were used in subsequent analyses (Menezes-Martins et al., 2005; Utine et al., 2008; Blaschke et al., 2011; Sancho-Tello et al., 2011; Feris-Iglesias et al., 2014; Papan et al., 2018; Amin et al., 2019; Tchatchouang et al., 2019).

### Characteristics of the included studies

**Table 1** summarizes the characteristics of the eight eligible studies (Menezes-Martins et al., 2005; Utine et al., 2008; Blaschke et al., 2011; Sancho-Tello et al., 2011; Feris-Iglesias et al., 2014; Papan et al., 2018; Amin et al., 2019; Tchatchouang et al., 2019). Four studies were conducted in high-income countries (Utine et al., 2008; Blaschke et al., 2011; Sancho-Tello et al., 2011; Papan et al., 2018), while four others were conducted in low- to middle-income countries (Menezes-Martins et al., 2005; Utine et al., 2008; Feris-Iglesias et al., 2014; Tchatchouang et al., 2019). The majority of studies were conducted on children (age <18 years) (Menezes-Martins et al., 2005; Utine et al., 2008; Blaschke et al., 2011; Feris-Iglesias et al., 2014; Papan et al., 2018; Amin et al., 2019), one on adults (Sancho-Tello et al., 2011), and one study did not report ages (Tchatchouang et al., 2019). Eight studies included 12 datasets. Of the 12 datasets, eight studies involving 424 samples assessed the accuracy of NAAT for SA detection (Menezes-Martins et al., 2005; Utine et al., 2008; Blaschke et al., 2011; Sancho-Tello et al., 2011; Feris-Iglesias et al., 2014; Papan et al., 2018; Amin et al., 2019; Tchatchouang et al., 2019), while four studies involving 317 samples assessed the accuracy of NAAT for MRSA detection (Menezes-Martins et al., 2005; Blaschke et al., 2011; Feris-Iglesias et al., 2014; Amin et al., 2019). Across the included studies, the total number of samples submitted for diagnostic evaluation ranged from 6 to 112, with a median value of 50. All experimental procedures were performed in tertiary care hospitals or a reference laboratory.

**Table 1:** Baseline characteristics of included studies.

First author	YOP	Country	Study period	Age (yrs)	Setting	Multi invol	Pros enrol	NOP	Patients selection	Specimen condition	Total PF Sample	NAAT specifics	Reported feature
Amin	2019	Iran	Mar 2018 – Sep 2018	<16	TCC	Yes	Yes	105	Convenience	Fresh	105	PCR	Empyema
Blaschke	2011	USA	Jan 2009 – Dec 2009	<18	TCC	No	No	63	Convenience	Fresh/Frozen	63	Nested PCR	PPE
Feris-Iglesias	2014	Dominican Republic	Jul 2009 – Jun 2011	<15	TCC	No	No	121	Convenience	Fresh/Frozen	112	PCR	PE
Menezes-Martins	2005	Brazil	NM	≤12	TCC	No	No	37	Consecutive	Fresh	37	PCR	Empyema
Papan	2018	Germany	Mar 2014 – Nov 2015	<2	RL	No	Yes	6	Convenience	Fresh	6	Unyvero mPCR	Pneumonia
Sancho-Tello	2011	Spain	Jul 2010 – Nov 2010	<65	TCC	No	No	7	Convenience	Fresh/Frozen	7	SeptiFast	Empyema
Tchatchouang	2019	Cameroon	Jan 2017 – Jan 2018	≥18	TCC	No	Yes	67	Convenience	Fresh/Frozen	67	RT-PCR	LRTI
Utine	2008	Turkey	2001 - 2003	<18	RL	No	No	28	Consecutive	Fresh/Frozen	28	PCR	PPE

LRTI, lower respiratory tract infection; mPCR, multiplex PCR; multi invol, multicenter involvement; NAAT, nucleic acid amplification tests; NM, not mentioned; NOP, number of patients; PCR, polymerase chain reaction; PE, pleural effusion; PF, pleural fluid; PPE, parapneumonic effusion; pros enrol, prospective enrollment; RL, reference laboratory; RT-PCR, reverse transcriptase PCR; TCC, tertiary care center; YOP, year of publication; yrs, years.

### Quality appraisal

**Figure 2:**
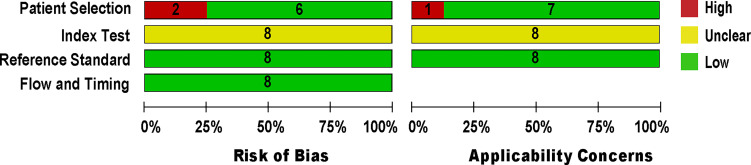
Methodological quality and risk of bias assessment of the eligible studies. The number of studies included in each domain is displayed.

The methodological quality of eligible studies was determined using QUADAS-2 (*see* **Figure 2**). Two studies (Feris-Iglesias et al., 2014; Papan et al., 2018) revealed a high risk of bias in the patient selection domain (*see* **Supplementary Figure S1**) due to specimen handling errors. The risk of bias in the index test domain was unclear for all studies because the studies did not report on index test blinding (Menezes-Martins et al., 2005; Utine et al., 2008; Blaschke et al., 2011; Sancho-Tello et al., 2011; Feris-Iglesias et al., 2014; Papan et al., 2018; Amin et al., 2019; Tchatchouang et al., 2019). The applicability concern in the index test domain was considered unclear due to the lack of a globally accepted index test protocol. The reference standard domain was supposedly at low risk of bias, as NAAT used pre-established binary response investigation criteria. All studies’ reference standards were performed in either a tertiary care center or a reference laboratory; thus, we expect operator error bias to be of low concern. Subsequently, the risk of bias in the flow and timing domain was not questioned because both index tests and reference standards were performed on the same samples. All articles met the criteria for the three domains of applicability concerns because most studies used pleural fluid samples from patients suspected of having empyema, which showed a low risk of bias.

### Summary estimates

**Figure 3:**
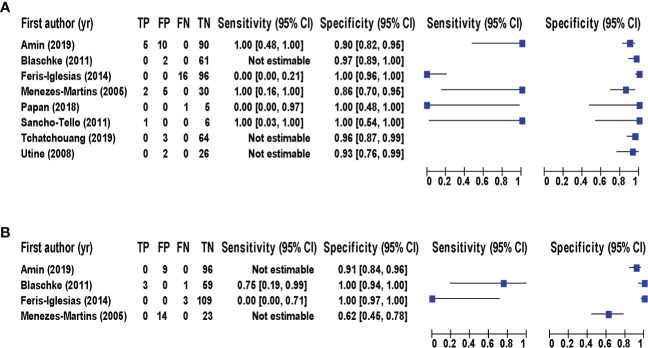
Forest plot for detection of **(A)** SA and **(B)** MRSA. The square stands for the estimated sensitivity and specificity of a particular study, and the black line represents its 95% confidence interval. CI, confidence interval; FN, false negative; FP, false positive; TN, true negative; TP, true positive; yr, year.

**Figure 4:**
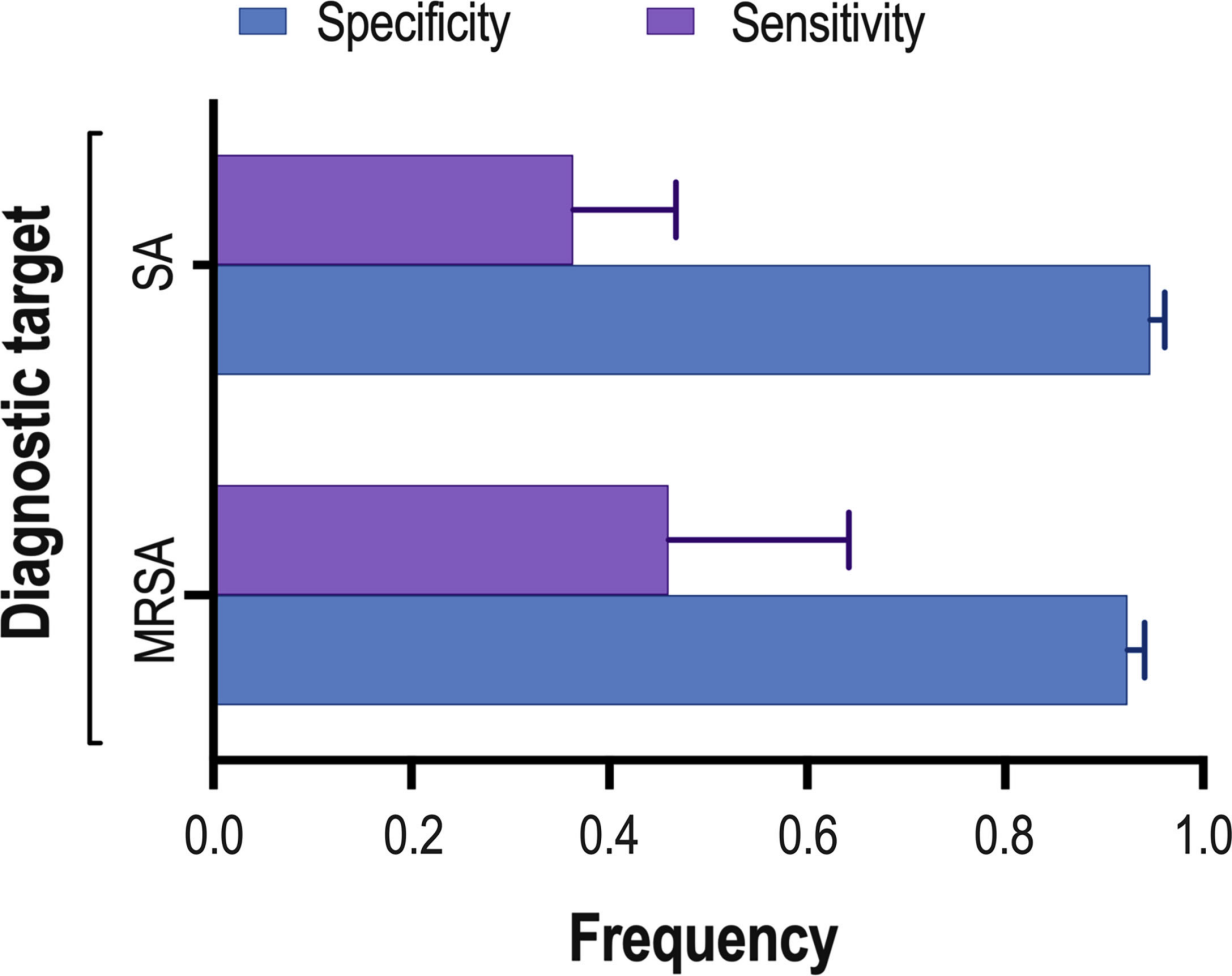
Summary of NAAT’s pooled sensitivity and specificity. The lines at the top of each horizontal bar indicate the standard error. : MRSA, methicillin-resistant *Staphylococcus aureus*; SA, *Staphylococcus aureus*.

**Figure 5:**
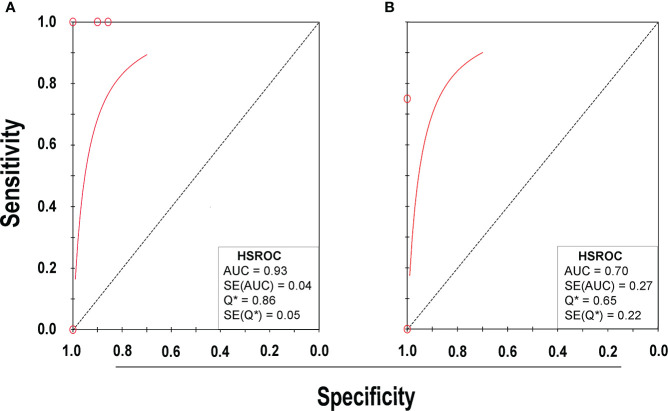
HSROC plot of NAAT for **(A)** SA and **(B)** MRSA detection. Red circles indicate the data point from each investigation, the solid red line represents the HSROC curve, and the diagonal line represents line of symmetry. AUC, area under the curve; HSROC, hierarchical summary receiver operating characteristic; Q*, an index defined by the point on the HSROC curve; SE (Q*), Q* index standard error.

Eight studies (Menezes-Martins et al., 2005; Utine et al., 2008; Blaschke et al., 2011; Sancho-Tello et al., 2011; Feris-Iglesias et al., 2014; Papan et al., 2018; Amin et al., 2019; Tchatchouang et al., 2019) consisting of a total of 424 samples met the inclusion criteria for comparing NAAT with a microbiological culture for SA detection in suspected empyema patients. The NAAT’s detection sensitivity for SA ranged from 0.0 (95% CI 0.00–0.21) to 1.00 (95% CI 0.48–1.00), while the specificity ranged from 0.86 (95% CI 0.70–0.95) to 1.0 (95% CI 0.96–1.00) (**Figure 3A**). The pooled sensitivity and specificity of NAAT for identification of SA were 0.35 (95% CI 0.19–0.55) and 0.95 (95% CI 0.92–0.97), respectively (**Figure 4**). The pooled positive likelihood ratio (PLR) for NAAT was 7.92 (95% CI 4.98–12.59), and the pooled negative likelihood ratio (NLR) for NAAT was 0.44 (95% CI 0.14–1.46). Additionally, the pooled DOR of NAAT was 24.03 (95% CI 6.59–87.61). The DOR (24.03 >1) indicated that NAAT was effective in our study. The statistical values for *I* ^2^ sensitivity and specificity were 74.9% and 65.0%, respectively, indicating substantial heterogeneity. The area under the curve (AUC) of the HSROC was 0.93 (95% CI 0.88–0.97), indicating overall justifiable diagnostic validity (**Figure 5A**).

With regard to MRSA detection (Menezes-Martins et al., 2005; Blaschke et al., 2011; Feris-Iglesias et al., 2014; Amin et al., 2019), a total of four studies comprising 317 samples evaluated the accuracy of NAAT against a microbiological culture reference standard. The sensitivity of NAAT for identification of MRSA ranged from 0.0 (95% CI 0.00–0.71) to 0.75 (95% CI 0.19–0.99), while the specificity ranged from 0.62 (95% CI 0.45–0.76) to 1.00 (95% CI 0.97–1.00), respectively (**Figure 3B**). The pooled sensitivity and specificity of NAAT for identification of MRSA were 0.45 (95% CI 0.15–0.78) and 0.93 (95% CI 0.89–0.95), respectively (**Figure 4**). The pooled PLR for NAAT was 10.06 (95% CI 1.49–67.69), and the pooled NLR for NAAT was 0.69 (95% CI 0.41–1.15). Additionally, the pooled DOR of NAAT was 27.18 (95% CI 2.97–248.6). The DOR (27.18 >1) indicated that the NAAT was effective in this study. The *I* ^2^ statistical scores for MRSA identification sensitivity and specificity were 13.7% and 94.4%, respectively, indicating mild to considerable heterogeneity. The AUC of HSROC for MRSA was 0.70 (95% CI 0.43–0.96), indicating that the diagnostic validity was overall acceptable (**Figure 5B**).

### Diagnostic accuracy of in-house vs commercial tests

**Table 2** summarizes the diagnostic accuracy of research findings based on different NAA tests. The pooled summary estimates of the in-house NAA tests for detecting SA (sensitivity: 0.33 (95% CI 0.16–0.54), specificity: 0.94 (95% CI 0.91–0.96), PLR: 7.85 (95% CI 4.82–12.78), NLR: 0.37 (95% CI 0.04–3.73), DOR: 29.54 (95% CI 6.23–140.0) and AUC 0.94 (95% CI 0.89–0.97) ) were slightly lower than those for MRSA (sensitivity: 0.45 (95% CI 0.15–0.78), specificity: 0.93 (95% CI 0.89–0.95), PLR: 10.06 (95% CI 1.49–67.69), NLR: 0.69 (95% CI 0.41–1.15), DOR: 27.18 (95% CI 2.97–248.6) and AUC 0.7 (95% CI 0.43–0.97) ). The *I* ^2^ statistical scores for SA in-house tests identification sensitivity and specificity were 84.4% and 78.4%, respectively, indicating substantial heterogeneity. However, when the *I* ^2^ statistical scores for SA commercial tests were evaluated, both identification sensitivity and specificity were 0.0%, indicating mild heterogeneity. The *I* ^2^ statistical scores for MRSA in-house tests were the same as overall detection sensitivity and specificity because all MRSA studies were in-house tests. For both SA and MRSA, the pooled summary estimates of conventional PCR are shown in **Table 2**. The *I* ^2^ heterogeneity scores for SA PCR tests identification sensitivity and specificity were 88.2% and 82.7%, respectively, indicating substantial heterogeneity. Whereas for MRSA PCR tests, the *I* ^2^ heterogeneity scores for identification sensitivity and specificity were 0.0% and 95.4%, respectively, suggesting mild to considerable heterogeneity. In comparison to other tests, PCR was consistently used in NAAT studies to detect staphylococcal empyema in pleural fluids.

**Table 2:** Subgroup analysis of studies based on different NAA tests.

Diagnostic target against culture reference standard	Subgroup	NAAT methods	NOS	First author (YOP )	Sensitivity (95% CI)	Specificity(95% CI)	PLR (95% CI)	NLR (95% CI)	DOR (95% CI)	AUC (95% CI)
SA	In-house		5		0.33 (0.16-0.54)	0.94 (0.91-0.96)	7.85 (4.82-12.78)	0.33 (0.02-6.82)	29.39 (5.49-157.2)	94 (89-97)
		PCR	4	Amin [2019] Feris-Iglesias [2014]); Menezes-Martins [2005] Utine [2008]	0.32 (0.15-0.54)	0.93 (0.90-0.96)	7.62 (4.63-12.53)	0.33 (0.02-6.82)	29.39 (5.49-157.2)	94 (89-98)
		Nested PCR	1	Blaschke [2011]	0.50 (0.0-1.00)	0.97 (0.89-1.00)	15.75 (1.45-171.5)	0.52 (0.07-3.69)	30.5 (0.47-1964.5)	–
	Commercial		3		0.50 (0.07-0.93)	0.96 (0.89-0.99)	8.55 (1.86-39.28)	0.63 (0.31-1.32)	15.14 (1.48-155.28)	97 (87-99)
		Unyvero mPCR	1	Papan [2018]	0.25 (0.0-0.94)	1.00 (0.48-1.00)	4.0 (0.18-88.7)	0.72 (0.32-1.68)	5.5 (0.13-236.4)	–
		SeptiFast	1	Sancho-Tello [2011]	1.00 (0.25-1.00)	1.00 (0.54-1.00)	10.5 (0.65-170.7)	0.27 (0.02-2.3)	39.0 (0.53-2883.6)	–
		RT-PCR	1	Tchatchouang [2019]	0.50 (0.0-1.00)	0.95 (0.92-0.97)	7.92 (4.98-12.59)	0.52 (0.07-3.72)	21.3 (0.36-1271.1)	–
MRSA	In-house		4		0.32 (0.15-0.54)	0.94 (0.91-0.97)	7.89 (4.8-12.99)	0.33 (0.02-6.83)	33.72 (6.31-180.3)	94 (90-99)
		PCR	3	Amin [2019]; Feris-Iglesias [2014] Menezes-Martins [2005]	0.25 (0.19-0.71)	0.93 (0.87-0.94)	4.98 (0.86-28.96)	0.79 (0.52-1.2)	27.18 (2.97-248.6)	60 (31-90)
		Nested PCR	1	Blaschke [2011]	0.75 (0.19-0.99)	1.00 (0.94-1.00)	84 (5.01-1408.2)	0.3 (0.08-1.15)	277.7 (9.48-8133.2)	–

-, not estimable; AUC, area under the curve; CI, confidence interval; DOR, diagnostic odds ratio; mPCR, multiplex PCR; MRSA, methicillin-resistant S. aureus; NAAT, nucleic acid amplification tests; NLR, negative likelihood ratio; NOS, number of studies; PCR, polymerase chain reaction; PLR, positive likelihood ratio; qPCR, quantitative PCR; RT-PCR, reverse transcriptase PCR; SA, Staphylococcus aureus; YOP, year of publication.

### Meta-regression and subgroup analysis

A meta-regression assessment on pre-specified subgroups was used to analyze the possible source of heterogeneity. The results of meta-regression analysis suggested that country (developing *vs* developed), setting (tertiary care center *vs* reference laboratory), study design (prospective *vs* others), patient selection (consecutive *vs* convenience), and sample condition (fresh *vs* frozen) were not significant sources of heterogeneity (meta-regression *P* = 0.66, *P* = 0.46, *P* = 0.98, *P* = 0.68, and *P* = 0.79, respectively) (*see* **Supplementary Figure S2**).

### Publication bias

Deek’s funnel plot asymmetry test was utilized to evaluate publication bias. This study found no evidence of significant publication bias (*P* = 0.85), indicating symmetry in the data and a low probability of publication bias (*see* **Supplementary Figure S3**).

## Discussion

Identification of the causative organism and effective antimicrobial therapy are critical in providing definitive therapy for staphylococcal empyema, which has been linked to a poor prognosis and higher mortality (Tsai et al., 2019; Kanellakis et al., 2022). However, the numerous case definitions and reference standards used in the different studies makes comparison of research findings difficult and limits disease management. Therefore, it is critical to identify SA and resistance markers in patients with suspected empyema as soon as possible, as a prompt intervention may greatly boost overall survival rates and reduce hospital burden. In the present study, the sensitivity and specificity of various NAA tests were evaluated against the currently most reliable culture reference standard. Based on our findings, we discovered that the pooled summary estimates of NAA tests for identifying SA and MRSA in pleural fluid were lower when using microbiological culture as a reference standard. However, when the total number of detections was taken into account, NAAT clearly outperformed microbiological culture (**Figure 3**). Thus, the use of NAAT in conjunction with microbiological culture should be considered, since it could lead to improved management of staphylococcal empyema.

To the best of our knowledge, this is the first meta-analysis to assess NAAT’s ability to detect staphylococcal empyema in clinically suspected patients by analyzing pleural fluid samples. The findings of this study suggest that that NAAT overall summary estimates for SA detection were comparable to MRSA, which is consistent with independent studies conducted in pleural fluid by other investigators (Le Monnier et al., 2006; Blaschke et al., 2013). Studies by Blaschke et al. (2013) and Le Monnier et al. (2006) demonstrated that molecular testing can provide detailed information about the etiology and epidemiology of empyema and other serious infections, particularly in culture-negative cases.

In this study, we noticed that NAAT identified more agents than microbiological cultures overall, which could be attributed to the fact that NAAT detects pathogens regardless of viability. Therefore, we recommend using NAAT in conjunction with culture to diagnose staphylococcal empyema because it provides timely results and can detect minute traces of bacterial DNA regardless of its living status. Although a positive test is not diagnostic of a diseased state, a negative result quickly and effectively rules it out. While previous systematic reviews have found that NAAT has a higher diagnostic value for MSSA and MRSA detection in the lower respiratory tract (LRT) and blood specimens (Chen et al., 2021; Chen et al., 2021), none of these reviews focused solely on pleural fluid for the definitive diagnosis of staphylococcal empyema. The accuracy of NAAT for detecting SA and MRSA was lower than that of microbiological culture in our study, which could be attributed to factors such as the study’s smaller sample size, different DNA extraction techniques, inhibitors in pleural fluid, and reaction material quality.

NAAT subgroup analysis revealed that in-house tests for detection of both SA and MRSA were comparable to overall diagnostic accuracy of NAAT. The PLR for the in-house test was consistently >7, implying that patients with staphylococcal empyema are ~7 times more likely than patients without empyema to be NAA test positive. It should be noted that, unlike SA, no commercial NAAT data for MRSA subgroups analysis was available to allow for detailed comparison because all MRSA studies were in-house tests. PCR was consistently used in NAAT studies to detect staphylococcal empyema, and septi*F*ast demonstrated the highest diagnostic accuracy in pleural fluid. In this study, countries, settings, study design, patient selection, and sample conditions were not identified as significant contributors to heterogeneity.

Our systematic review’s strengths include a comprehensive search strategy that identified all relevant studies from five of the most popular and widely used large databases, with no language restrictions. The searches were conducted systematically, and at least two authors reviewed the titles and abstracts of all studies. The articles included in this systematic review reflect the authors’ collective opinion following group discussion. This study adhered to the PRISMA guidelines for systematic reviews and used the QUADAS-2 tool to assess the methodological quality of the included studies. The subsequent analysis excluded studies that did not follow specified guidelines for diagnosing staphylococcal empyema. This study used a precise microbiological culture reference standard, a bivariate random-effects model for data manipulation, and meta-regression analysis on predefined subgroups to interpret NAAT accuracy. Furthermore, studies involving pre-enrichment steps prior to molecular testing and studies involving nucleic acid amplification with sequencing, which may tend to overstate the index test’s diagnostic performance, were excluded.

This study has some limitations that should be taken into consideration. We are likely to have missed a few important studies through systematic literature searches across databases. The subgroup and meta-regression analyses revealed that variables such as the NAA techniques and standard tests were likely causes of the heterogeneity. We were unable to address the impact of variables such as sample volume, processing steps, amplification protocols, expertise with NAA tests, and laboratory infrastructure on NAA test accuracy due to a high level of variability in these factors and reporting of these factors in the studies. In addition, this meta-analysis was constrained due to a limited number of studies evaluating the accuracy of molecular tests in pleural fluid, particularly among adults, and should be interpreted with caution. Finally, as with any systematic review, limitations due to potential publication bias were a cause for concern.

## Conclusions

The findings of this study suggest that NAA tests may not have adequate diagnostic accuracy to replace microbial cultures in diagnosing staphylococcal empyema. However, since NAA tests have a faster turnaround time and can detect dead pathogens, they should be used in conjunction with microbiological culture. Given the scarcity of data on staphylococcal strains, a thorough investigation involving a larger number of prospective studies would be worthwhile to fully validate the clinical outcomes associated with NAAT’s utility. Furthermore, future research should investigate additional measures, such as NAAT’s impact on cost-effectiveness, decreased hospitalizations, and adverse antimicrobial effects, to facilitate therapeutic adaptations.

## Author contributions

KC and SCO conceptualized the study. SCO, KC, YY, and SA performed the literature search, analyzed data, and drafted the manuscript. KC, AAM, MN, Y-JS, CS, GW, and C-LD reviewed and edited the manuscript. All authors read and approved the final manuscript.

## Funding

This study was funded in part by the National Science Foundation of China (Grant No. 82150410452) and the Doctoral research Fund to SCO and KC.

## Conflict of interest

The authors declare that the research was conducted in the absence of any commercial or financial relationships that could be construed as a potential conflict of interest.

## Publisher’s note

All claims expressed in this article are solely those of the authors and do not necessarily represent those of their affiliated organizations, or those of the publisher, the editors and the reviewers. Any product that may be evaluated in this article, or claim that may be made by its manufacturer, is not guaranteed or endorsed by the publisher.
