# Supplementary material for: Clinical relevance of molecular testing methods in the diagnosis and guidance of therapy in patients with staphylococcal empyema: a systematic review and meta-analysis
Source: Front Cell Infect Microbiol. 2022 Jul 29;12:758833. doi: 10.3389/fcimb.2022.758833 (PMC9372472; doi:10.3389/fcimb.2022.758833)
Supplement: Supplementary file 1 [file DataSheet_1.docx]

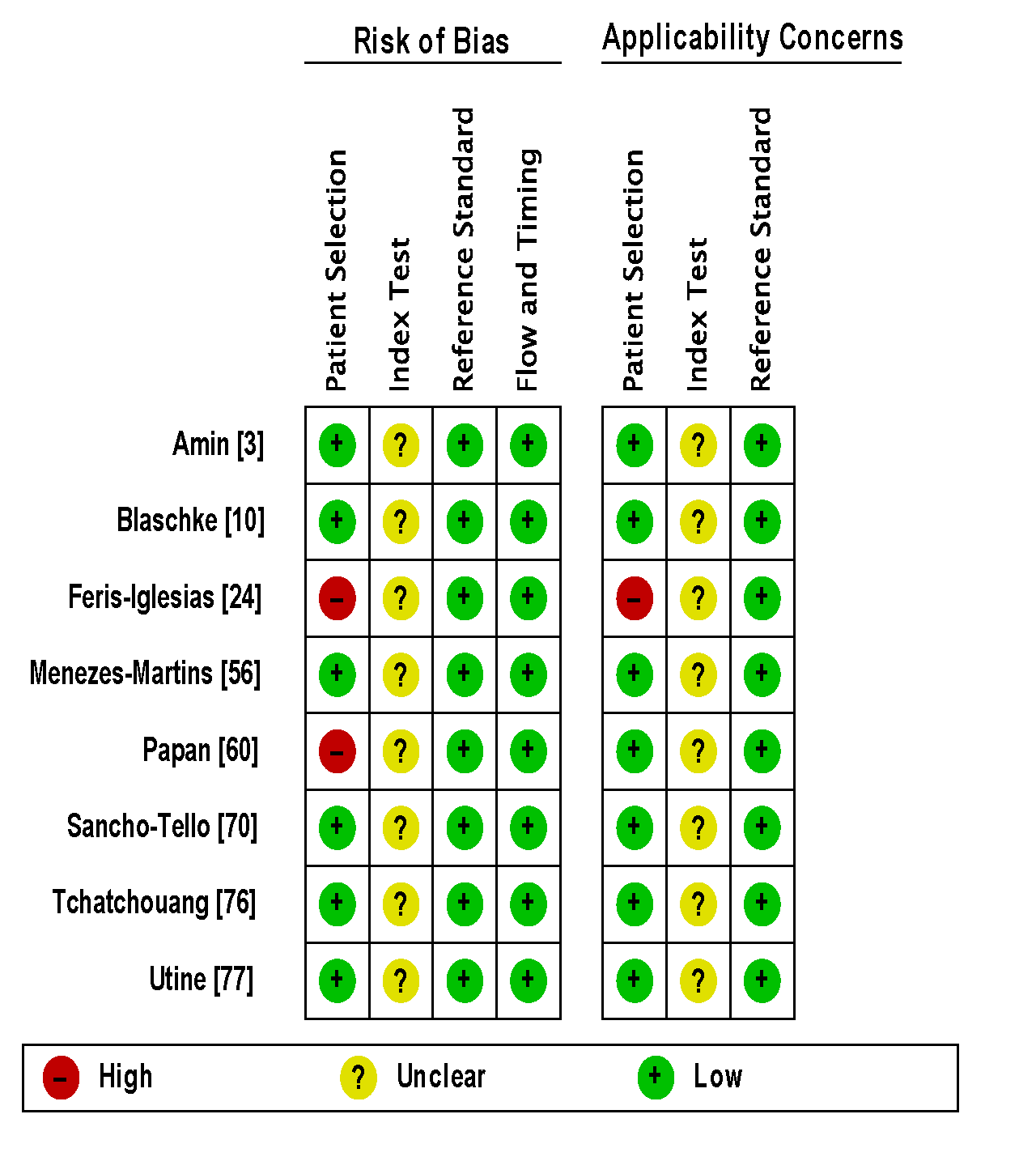


**Supplementary Fig. 1** Individual study methodological quality and risk of bias assessment using the QUADAS-2 tool.

(A) Meta-regression analysis by country status


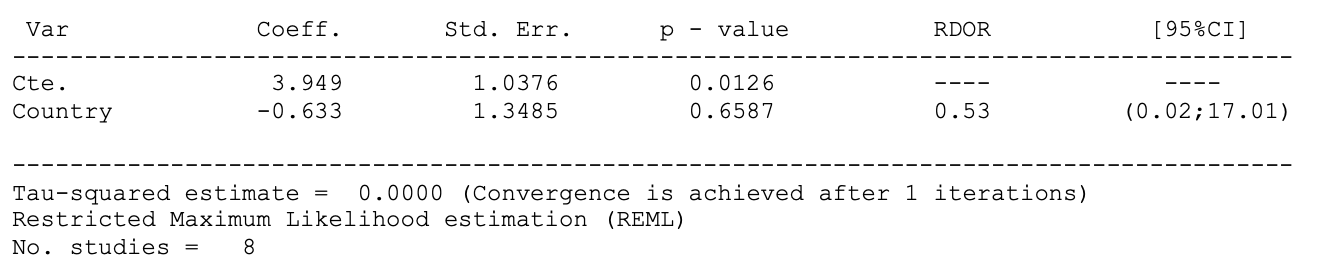


(B) Meta-regression analysis by setting


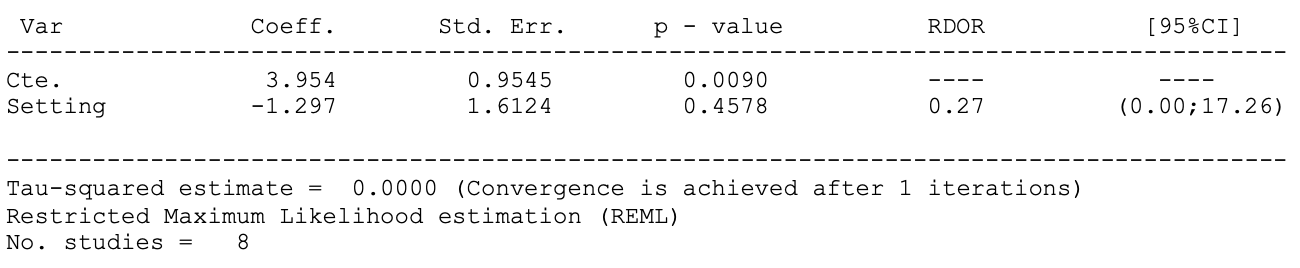


(C) Meta-regression analysis by study design


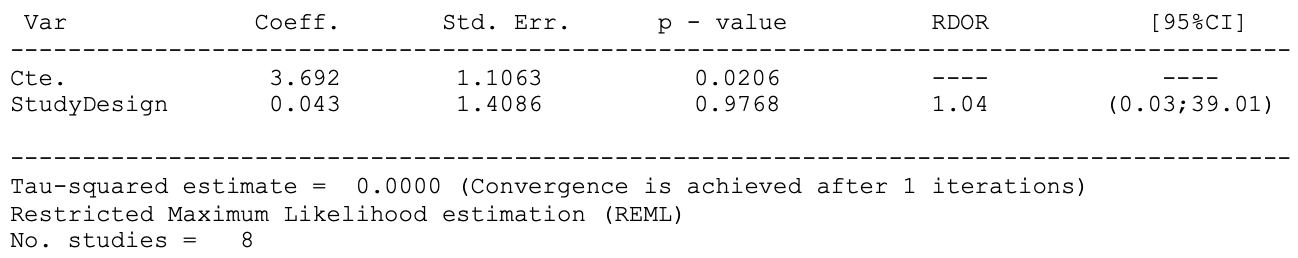


(D) Meta-regression analysis by patient selection


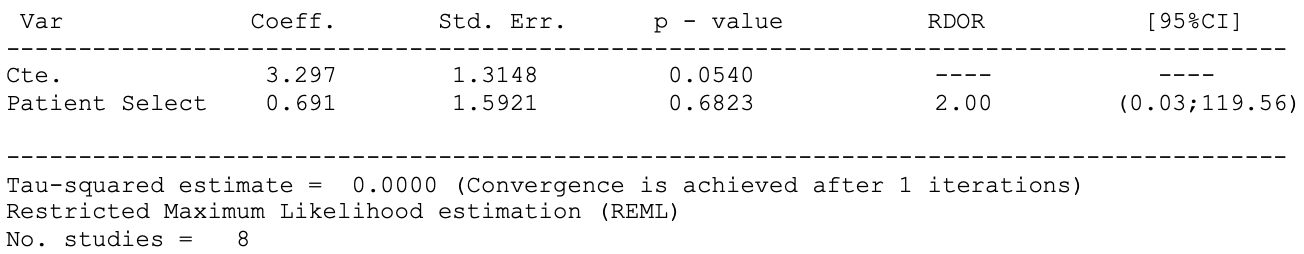


(E) Meta-regression analysis by sample conditions


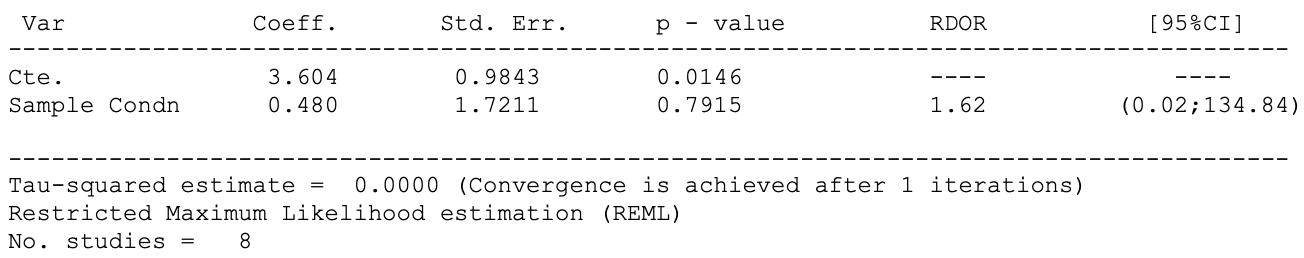


**Supplementary Fig. 2** Meta-regression analysis of predefined subgroups based on (A) country status, (B) setting, (C) study design, (D) patient selection, and (E) sample conditions. *Abbreviations*: CI, confidence interval; coeff., coefficient; Condn, conditions; cte, conditional tail expectation; No., number; p-value, probability value; RDOR, relative diagnostic odds ratio; REML, restricted maximum likelihood estimation; Select, selection; Std. Err., standard error; StudyDesign, study design; Var, variable.


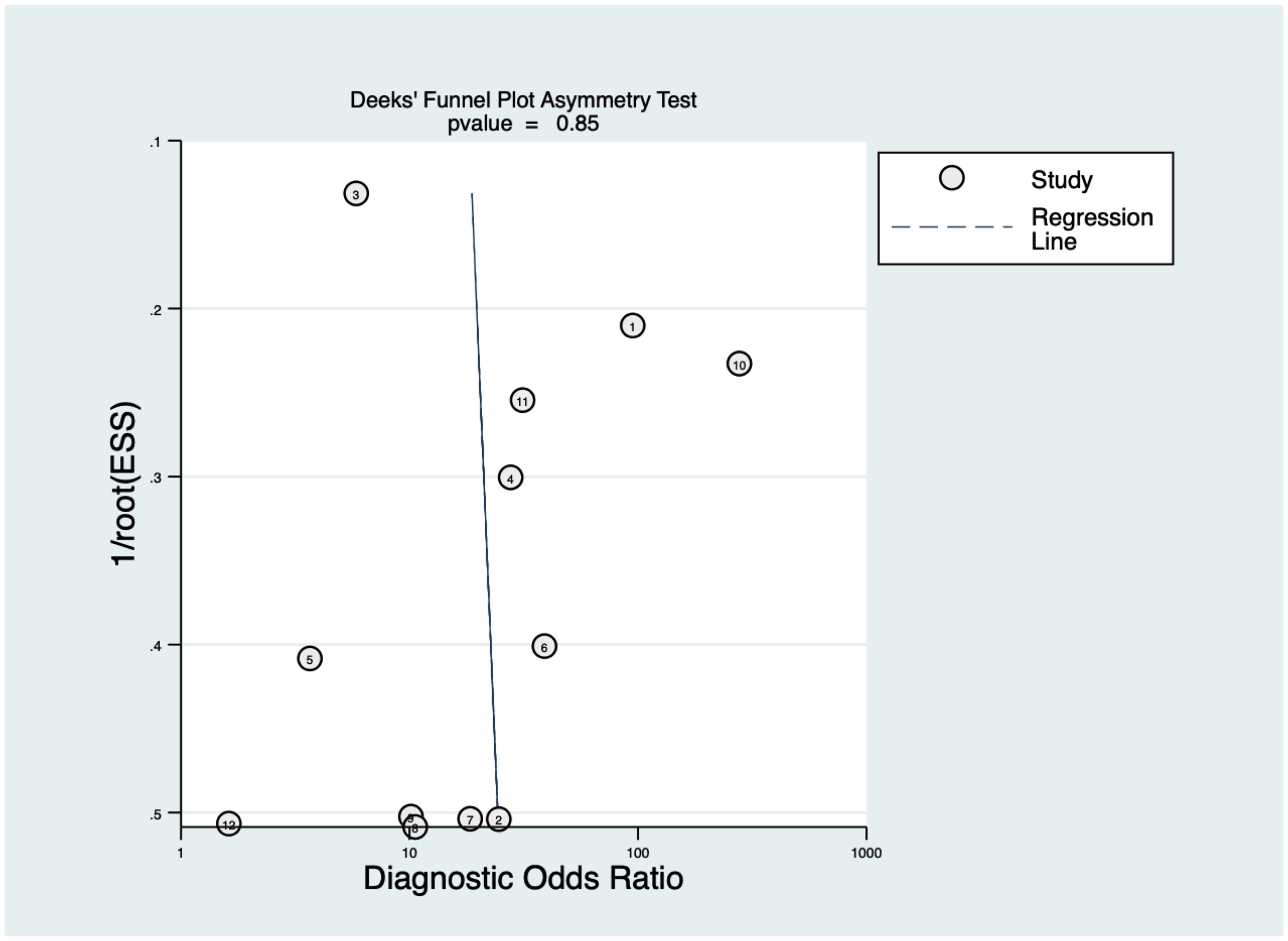


**Supplementary Fig. 3** Deek’s funnel plot asymmetry test for publication bias. The numbers 1–12 represent the datasets used in our research for the diagnosis of MSSA and MRSA. It should be noted that because some of the datasets come from the same studies, the overlapping numbers may not be displayed.

**Supplementary Table 1** Details of all the publications that were deemed relevant, and hence, fully screened by the first two authors (*n* = 83; i.e., 76 following duplicates removal + 7 via reference searches). Reasons why studies have been excluded are given (*n* = 72). Studies included in our meta-analysis are shaded with grey color (*n* = 8).

| First author (reference) | Inclusion (I)  /Exclusion (E) | Reasons |
| --- | --- | --- |
| Akusheva (1) | E | Non-English publication |
| Alam (2) | E | Pericardial effusion associated with TB |
| Amin (3) | I |  |
| Ardura (4) | E | No sensitivity and specificity / not sufficient data |
| Atkinson (5) | E | Review article |
| Bahrain (6) | E | Associated with bacterial endocarditis |
| Bateman (7) | E | Conference abstract |
| Beckham (8) | E | Review article |
| Blaschke (9) | E | Letter to the editor |
| Blaschke (10) | I |  |
| Camoez (11) | E | Molecular characterization of MRSA-ST398 clone |
| Ceyhan (12) | E | Serotype distribution of *S. pneumonia* in children |
| Chacon-Cruz (13) | E | No sensitivity and specificity / not sufficient data |
| Chandak (14) | E | Conference abstract |
| Chen (15) | E | Microbiome analysis in pleural empyema |
| Chow (16) | E | *Helcococcus* spp. from a patient with urosepsis |
| Cihanoglu (17) | E | No sensitivity and specificity / not sufficient data |
| Cremades (18) | E | Culture comparison with 16S rRNA PCR with sequencing |
| Delerme (19) | E | Conference abstract |
| Derber & Troy (20) | E | Review article |
| Ding (21) | E | Case report |
| Doudoulakakis (22) | E | No sensitivity and specificity / not sufficient data |
| Dwari (23) | E | No sensitivity and specificity / not sufficient data |
| Feris-Iglesias (24) | I |  |
| Foissac (25) | E | Case report |
| Franchetti (26) | E | No sensitivity and specificity / not sufficient data |
| Gareca Perales (27) | E | No sensitivity and specificity / not sufficient data |
| Garza-González (28) | E | Determine drug-resistance in coagulase-negative staphylococcal strains |
| Gaudelus (29) | E | Non-English publication |
| Gollomp (30) | E | Culture comparison with 16S rRNA PCR with sequencing |
| Gomes (31) | E | No sensitivity and specificity / not sufficient data |
| Gonzalez (32) | E | No sensitivity and specificity / not sufficient data |
| Gronthoud (33) | E | Case report |
| Guimaraes (34) | E | Identify cell-free DNA in staphylococcal bacteremia |
| Harris (35) | E | Guidelines for management of pneumonia in children |
| Hatcher (36) | E | *S. aureus* nasal carriage among industrial hog workers |
| Henares (37) | E | 2×2 contingency table could not be obtained |
| Hosseini (38) | E | Case report |
| ICTRP (39) | E | Clinical trial that is unrelated to the current study |
| Insa (40) | E | Culture comparison with 16S rRNA PCR with sequencing |
| Johansson (41) | E | Culture comparison with 16S rDNA PCR with sequencing |
| Kacprzak (42) | E | Case reports |
| Kaewbumrung (43) | E | Antibacterial activity of plumbagin derivative |
| Kanishani (44) | E | No sensitivity and specificity / not sufficient data |
| Karbuz (45) | E | Evaluation of AST & virulence factor on isolated strains |
| Kerem (46) | E | Meeting abstract |
| Krenke (47) | E | No staphylococcal strains obtained from PF |
| Le Monnier (48) | E | Culture comparison with 16S rRNA PCR with sequencing |
| Li (49) | E | No sensitivity and specificity / not sufficient data |
| Lochindarat (50) | E | No sensitivity and specificity / not sufficient data |
| Mahdiyoun (51) | E | Determination of aminoglycoside-resistance in MRSA isolates |
| Mahoudeau (52) | E | Isolation of *Staphylococcus intermedius* |
| Malik (53) | E | No comparative evaluation with molecular tests |
| Maskell (54) | E | Culture comparison with 16S rRNA PCR with sequencing |
| McBride (55) | E | Review article |
| Menezes-Martins (56) | I |  |
| Meyer Sauteur (57) | E | No sensitivity and specificity / not sufficient data |
| Micek (58) | E | Case reports |
| Mutimer (59) | E | Sepsis in liver transplant recipient |
| Papan (60) | I |  |
| Park (61) | E | Determines colonization density of upper respiratory tract |
| Pereira (62) | E | Detection of oxacillin resistance from the isolated strains |
| Pernica (63) | E | No positive staphylococcal strains |
| Peterson (64) | E | Animal studies |
| Psallidas (65) | E | Evaluation of ultrasound-guided pleural biopsies |
| Rao (66) | E | Conference abstract |
| Rozenbaum (67) | E | Case report |
| Saglani (68) | E | Culture comparison with 16S rRNA PCR with sequencing |
| Samra (69) | E | Case report |
| Sancho-Tello (70) | I |  |
| Sárvári (71) | E | Isolation of *S. moorei* |
| Shah (72) | E | Editorial commentary |
| Skouras (73) | E | Review article |
| Sohn (74) | E | Evaluation of synovial fluid ADA level in TB arthritis |
| Sparo (75) | E | Characterization of enterocin from a corn silage |
| Tchatchouang (76) | I |  |
| Utine (77) | I |  |
| Vu-Thien (78) | E | Non-English publication |
| Wiersinga (79) | E | Guidelines on the management of CAP |
| Wrightson (80) | E | Conference abstract |
| Xirogianni (81) | E | Development of an assay |
| Zheng (82) | E | Detection of *S. pyogenes* in pediatric empyema |
| Zhu (83) | E | LAMP method for mycobacterium spp. detection |

*Abbreviations*: ADA, adenosine deaminase; AST, antibiotic sensitivity test; CAP, community acquired pneumonia; DNA, deoxyribonucleic acid; LAMP, loop-mediated isothermal amplification; MRSA, methicillin-resistant *Staphylococcus aureus*; PCR, polymerase chain reaction; PF, pleural fluid; rDNA, ribosomal DNA; rRNA, ribosomal ribonucleic acid; spp., species; TB, tuberculosis.

**References**

1. Akusheva DN, Khokhlova OE, Kamshilova VV, Motova AI, Peryanova OV, Upirova AA, et al. Community-acquired pneumonia in HIV-infected subjects: Microflora, antibiotic resistance: Dependence on the levels of CD4 lymphocytes. Med. Immunol. (Russ.). 2019;21(3):457-66.

2. Alam MT, Lashari MN, Ahmed S, Siddiqui AS, Aslam M. Frequency of culture positive tuberculosis in exudative pericardial effusion. Med. Forum Mon. 2013;24(4):38-41.

3. Amin M, Yousef Pour S, Navidifar T. Detection of the major bacterial pathogens among children suffering from empyema in Ahvaz city, Iran. J. Clin. Lab. Anal. 2019;33(4):e22855.

4. Ardura MI, Mejías A, Katz KS, Revell P, McCracken Jr GH, Sánchez PJ. Daptomycin therapy for invasive Gram-positive bacterial infections in children. Pediatr. Infect. Dis. J. 2007;26(12):1128-32.

5. Atkinson M, Yanney M, Stephenson T, Smyth A. Effective treatment strategies for paediatric community-acquired pneumonia. Expert Opin. Pharmacother. 2007;8(8):1091-101.

6. Bahrain M, Vasiliades M, Wolff M, Younus F. Five cases of bacterial endocarditis after furunculosis and the ongoing saga of community-acquired methicillin-resistant *Staphylococcus aureus* infections. Scand. J. Infect. Dis. 2006;38(8):702-7.

7. Bateman RM, Sharpe MD, Jagger JE, Ellis CG, Solé-Violán J, López-Rodríguez M, et al. 36th International symposium on intensive care and emergency medicine : Brussels, Belgium. 15-18 March 2016. Crit Care. 2016;20(Suppl 2):94.

8. Beckham JD, Tyler KL. Neuro-intensive care of patients with acute CNS infections. Neurotherapeutics. 2012;9(1):124-38.

9. Blaschke AJ, Byington CL, Ampofo K, Pavia AT, Heyrend C, Rankin SC, et al. Species-specific PCR improves detection of bacterial pathogens in parapneumonic empyema compared with 16SPCR and culture. Pediatr. Infect. Dis. J. 2013;32(3):302-3.

10. Blaschke AJ, Heyrend C, Byington CL, Obando I, Vazquez-Barba I, Doby EH, et al. Molecular analysis improves pathogen identification and epidemiologic study of pediatric parapneumonic empyema. Pediatr. Infect. Dis. J. 2011;30(4):289.

11. Camoez M, Sierra JM, Pujol M, Hornero A, Martin R, Domínguez MA. Prevalence and molecular characterization of methicillin-resistant *Staphylococcus aureus* ST398 resistant to tetracycline at a Spanish hospital over 12 Years. PLoS One. 2013;8(9).

12. Ceyhan M, Aykac K, Gurler N, Ozsurekci Y, Öksüz L, Altay Akısoglu Ö, et al. Serotype distribution of *Streptococcus pneumonia* in children with invasive disease in Turkey: 2015-2018. Hum. Vaccines Immunother. 2020;16(11):2773-8.

13. Chacon-Cruz E, Rivas-Landeros RM, Volker-Soberanes ML, Lopatynsky-Reyes EZ, Becka C, Alvelais-Palacios JA. 12 years active surveillance for pediatric pleural empyema in a Mexican hospital: effectiveness of pneumococcal 13-valent conjugate vaccine, and early emergence of methicillin-resistant *Staphylococcus aureus*. Ther. Adv. Infect. Dis. 2019;6:2049936119839312.

14. Chandak T, Ciofoaia G, Murati J, Alarcon S. 1034: Negative nasal PCR in community-acquired MRSA (CA-MRSA) necrotizing pneumonia with septic shock. Crit. Care Med. 2018;46(1):501.

15. Chen Z, Cheng H, Cai Z, Wei Q, Li J, Liang J, et al. Identification of microbiome etiology associated with drug resistance in pleural empyema. Front. Cell Infect. Microbiol. 2021;11:637018.

16. Chow SK, Clarridge JE, 3rd. Identification and clinical significance of Helcococcus species, with description of *Helcococcus seattlensis* sp. nov. from a patient with urosepsis. J Clin Microbiol. 2014;52(3):854-8.

17. Cihanoglu N, Adaleti R, Nakipoglu Y. Investigation of fibronectin binding protein (FBP) and panton valentine leukocidin (PVL) viulance factors in clinical methicillin sensitive and resistant *Staphylococcus aureus* strains. Clin. Lab. 2019;65(1).

18. Cremades R, Galiana A, Rodriguez JC, Santos A, Lopez P, Ruiz M, et al. Identification of bacterial DNA in noninfectious pleural fluid with a highly sensitive PCR method. Respiration. 2011;82(2):130-5.

19. Delerme A, Toocheck C, Karsies T, Watson J. Clinic utility of nasal Staphylococcus aureus screening by polymerase chain reaction (PCR) in pediatric pneumonia. TP93 TP093 NEW DEVELOPMENTS IN DIAGNOSTICS AND TREATMENTS OF PNEUMONIA: American Thoracic Society; 2021. p. A3876-A.

20. Derber CJ, Troy SB. Head and neck emergencies: Bacterial meningitis, encephalitis, brain abscess, upper airway obstruction, and jugular septic thrombophlebitis. Med. Clin. North Am. 2012;96(6):1107-26.

21. Ding Y, Steed LL, Batalis N. First reported case of disseminated *Microascus gracilis* infection in a lung transplant patient. IDCases. 2020;22.

22. Doudoulakakis AG, Bouras D, Drougka E, Kazantzi M, Michos A, Charisiadou A, et al. Community-associated *Staphylococcus aureus* pneumonia among Greek children: epidemiology, molecular characteristics, treatment, and outcome. Eur. J. Clin. Microbiol. Infect. Dis. 2016;35(7):1177-85.

23. Dwari AK, Jha S, Sarkar S, Misra S, Chakraborty S, Mandal A. A study of bacterial isolates and their sensitivity pattern to antibiotics in empyema thoracis cases in a tertiary care hospital. J. Evol. Med. Dent. Sci.. 2018;7(38):4978-81.

24. Feris-Iglesias J, Fernández J, Sánchez J, Pimenta F, Peña C, Coradin H, et al. Aetiology of paediatric pneumonia with effusion in the Dominican Republic and the potential impact of pneumococcal conjugate vaccines. Pneumonia (Nathan). 2014;4:8-15.

25. Foissac M, Bergon L, Vidal J, Cauquil P, Mainar A, Mourguet M. Pneumonia and pulmonary abscess due to legionella micdadei in an immunocompromised patient. Germs. 2019;9(2):89-94.

26. Franchetti L, Schumann DM, Tamm M, Jahn K, Stolz D. Multiplex bacterial polymerase chain reaction in a cohort of patients with pleural effusion. BMC Infect. Dis. 2020;20(1):1-10.

27. Gareca Perales J, Soleto Ortiz L, Loayza Mafayle R, Machuca Soto B, Hidalgo Flores L, López Montaño J, et al. Diagnosis of community-acquired pneumonia in hospitalized children: a multicenter experience in Bolivia. Pediatr. Infect. Dis. J. 2021;40(1):32-8.

28. Garza-González E, López D, Pezina C, Muruet W, Bocanegra-García V, Muñoz I, et al. Diversity of staphylococcal cassette chromosome mec structures in coagulase-negative staphylococci and relationship to drug resistance. J. Med. Microbiol. 2010;59(Pt 3):323-9.

29. Gaudelus J, Dubos F, Dommergues MA, Vu Thien H, Bingen E, Cohen R. Antibiotic treatment of child empyema: lessons from published studies and therapeutic options. Arch. Pediatr. 2008;15(Suppl. 2):S84-S92.

30. Gollomp K, Rankin SC, White C, Mattei P, Harris MC, Kilpatrick LE, et al. Broad‐range bacterial polymerase chain reaction in the microbiologic diagnosis of complicated pneumonia. J. Hosp. Med. 2012;7(1):8-13.

31. Gomes RT, Lyra TG, Alves NN, Caldas RM, Barberino MG, Nascimento-Carvalho CM. Methicillin-resistant and methicillin-susceptible community-acquired *Staphylococcus aureus* infection among children. Braz. J. Infect. Dis. 2013;17(5):573-8.

32. Gonzalez BE, Hulten KG, Dishop MK, Lamberth LB, Hammerman WA, Mason Jr EO, et al. Pulmonary manifestations in children with invasive community-acquired *Staphylococcus aureus* infection. Clin. Infect. Dis. 2005;41(5):583-90.

33. Gronthoud F, Hassan I, Newton P. Primary pyogenic ventriculitis caused by *Neisseria meningitidis*: case report and review of the literature. JMM Case Rep. 2017;4(1):e005078.

34. Guimaraes AO, Gutierrez J, Maskarinec SA, Cao Y, Hong K, Ruffin F, et al. Prognostic power of pathogen cell-free DNA in *Staphylococcus aureus* bacteremia. Open Forum Infect. Dis. 2019;6(4).

35. Harris M, Clark J, Coote N, Fletcher P, Harnden A, McKean M, et al. British Thoracic Society guidelines for the management of community acquired pneumonia in children: Update 2011. Thorax. 2011;66(SUPPL. 2):ii1-ii23.

36. Hatcher SM, Rhodes SM, Stewart JR, Silbergeld E, Pisanic N, Larsen J, et al. The prevalence of antibiotic-resistant *Staphylococcus aureus* nasal carriage among industrial hog operation workers, community residents, and children living in their households: North carolina, USA. Environ. Health Perspect. 2017;125(4):560-9.

37. Henares D, Brotons P, Buyse X, Latorre I, de Paz HD, Muñoz-Almagro C. Evaluation of the eazyplex MRSA assay for the rapid detection of *Staphylococcus aureus* in pleural and synovial fluid. Int. J. Infect. Dis. 2017;59:65-8.

38. Hosseini MJ, Fooladi AAI. Miliary tuberculosis with empyema, a case report. Jundishapur J. Microbiol. 2010;3(3):129-32.

39. ICTRP W. A study to assess the efficacy and safety of ceftobiprole medocaril compared to daptomycin in the treatment of *Staphylococcus aureus* bacteremia, including infective endocarditis. 2019(07).

40. Insa R, Marín M, Martín A, Martín-Rabadán P, Alcalá L, Cercenado E, et al. Systematic use of universal 16S rRNA gene polymerase chain reaction (PCR) and sequencing for processing pleural effusions improves conventional culture techniques. Medicine. 2012;91(2):103-10.

41. Johansson N, Vondracek M, Backman-Johansson C, Sköld MC, Andersson-Ydsten K, Hedlund J. The bacteriology in adult patients with pneumonia and parapneumonic effusions: increased yield with DNA sequencing method. Eur. J. Clin. Microbiol. Infect. Dis. 2019;38(2):297-304.

42. Kacprzak G, Majewski A, Kolodziej J, Rzechonek A, Gürlich R, Bobek V. New therapy of pleural empyema by deoxyribonuclease. Braz. J. Infect. Dis. 2013;17(1):90-3.

43. Kaewbumrung S, Panichayupakaranant P. Antibacterial activity of plumbagin derivative-rich Plumbago indica root extracts and chemical stability. Nat. Prod. Res. 2014;28(11):835-7.

44. Kanishani C, Shetty VA, Hampana S, Sharma RR, Shetty AK. Bacterial aetiology of community acquired pneumonia in a tertiary care hospital of southern India. J. Clin. Diagn. Res. 2020;14(2).

45. Karbuz A, Karahan ZC, Aldemir-Kocabaş B, Tekeli A, Özdemir H, Güriz H, et al. Evaluation of antimicrobial susceptibilities and virulence factors of *Staphylococcus aureus* strains isolated from community-acquired and health-care associated pediatric infections. Turk. J. Pediatr. 2017;59(4):395-403.

46. Kerem E. Complicated pneumonia in the developed world. Paediatr. Respir. Rev. 2010(11):S23-S4.

47. Krenke K, Sadowy E, Podsiadły E, Hryniewicz W, Demkow U, Kulus M. Etiology of parapneumonic effusion and pleural empyema in children. The role of conventional and molecular microbiological tests. Respir. Med. 2016;116:28-33.

48. Le Monnier A, Carbonnelle E, Zahar J-R, Le Bourgeois M, Abachin E, Quesne G, et al. Microbiological diagnosis of empyema in children: comparative evaluations by culture, polymerase chain reaction, and pneumococcal antigen detection in pleural fluids. Clin. Infect. Dis. 2006;42(8):1135-40.

49. Li S, Ning X, Song W, Dong F, Zheng Y, Chen Q, et al. Clinical and molecular characteristics of community-acquired methicillin-resistant *Staphylococcus aureus* infections in Chinese neonates. APMIS. 2015;123(1):28-36.

50. Lochindarat S, Teeratakulpisarn J, Warachit B, Chanta C, Thapa K, Gilbert GL, et al. Bacterial etiology of empyema thoracis and parapneumonic pleural effusion in Thai children aged less than 16 years. Southeast Asian J. Trop. Med. Public Health. 2014;45(2):442-54.

51. Mahdiyoun SM, Kazemian H, Ahanjan M, Houri H, Goudarzi M. Frequency of aminoglycoside-resistance genes in methicillin-resistant *Staphylococcus aureus* (MRSA) isolates from hospitalized patients. Jundishapur J. Microbiol. 2016;9(8):e35052.

52. Mahoudeau I, Delabranche X, Prevost G, Monteil H, Piemont Y. Frequency of isolation of *Staphylococcus intermedius* from humans. J. Clin. Microbiol. 1997;35(8):2153-4.

53. Malik M, Malik MI, Akhtar FK. Microbiological profile and antibiogram of lower respiratory tract infections at lahore general hospital, Lahore. J. Postgrad. Med. Inst. 2019;33(1):23-9.

54. Maskell NA, Batt S, Hedley EL, Davies CW, Gillespie SH, Davies RJ. The bacteriology of pleural infection by genetic and standard methods and its mortality significance. Am. J. Respir. Crit. Care Med. 2006;174(7):817-23.

55. McBride SC. Management of parapneumonic effusions in pediatrics: Current practice. J. Hosp. Med. 2008;3(3):263-70.

56. Menezes-Martins LF, Menezes-Martins JJ, Michaelsen VS, Aguiar BB, Ermel T, Machado DC. Diagnosis of parapneumonic pleural effusion by polymerase chain reaction in children. J. Pediatr. Surg. 2005;40(7):1106-10.

57. Meyer Sauteur PM, Burkhard A, Moehrlen U, Relly C, Kellenberger C, Ruoss K, et al. Pleural tap-guided antimicrobial treatment for pneumonia with parapneumonic effusion or pleural empyema in children: a single-center cohort study. J. Clin. Med. 2019;8(5).

58. Micek ST, Dunne M, Kollef MH. Pleuropulmonary complications of Panton-Valentine leukocidin-positive community-acquired methicillin-resistant *Staphylococcus aureus*: importance of treatment with antimicrobials inhibiting exotoxin production. Chest. 2005;128(4):2732-8.

59. Mutimer D, Mirza D, Shaw J, O'Donnell K, Elias E. Enhanced (cytomegalovirus) viral replication associated with septic bacterial complications in liver transplant recipients. Transplantation. 1997;63(10):1411-5.

60. Papan C, Meyer-Buehn M, Laniado G, Nicolai T, Griese M, Huebner J. Assessment of the multiplex PCR-based assay Unyvero pneumonia application for detection of bacterial pathogens and antibiotic resistance genes in children and neonates. Infection. 2018;46(2):189-96.

61. Park DE, Baggett HC, Howie SRC, Shi Q, Watson NL, Brooks WA, et al. Colonization density of the upper respiratory tract as a predictor of pneumonia-*Haemophilus influenzae*, *Moraxella catarrhalis*, *Staphylococcus aureus*, and *Pneumocystis jirovecii*. Clin. Infect. Dis. 2017;64(suppl_3):S328-s36.

62. Pereira VC, Martins A, de Souza Rugolo LM, de Lourdes Ribeiro de Souza da Cunha M. Detection of oxacillin resistance in *Staphylococcus aureus* isolated from the neonatal and pediatric units of a brazilian teaching hospital. Clin. Med. Pediatr. 2009;3:23-31.

63. Pernica JM, Moldovan I, Chan F, Slinger R. Real-time polymerase chain reaction for microbiological diagnosis of parapneumonic effusions in Canadian children. Can. J. Infect. Dis. Med. Microbiol. 2014;25(3):151-4.

64. Peterson GE, Silva SS, Amantéa SL, Miorelli P, Sanches P, Kulczynski J, et al. Accuracy of complement activation product levels to detect infected pleural effusion in rats. Pediatr. Pulmonol. 2017;52(6):757-62.

65. Psallidas I, Kanellakis NI, Bhatnagar R, Ravindran R, Yousuf A, Edey AJ, et al. A pilot feasibility study in establishing the role of ultrasound-guided pleural biopsies in pleural infection (the AUDIO study). Chest. 2018;154(4):766-72.

66. Rao H, Ericson J, Hollern K, O'Hara C. Pericardial and pleural effusion in an adolescent with Mycoplasma Pneumoniae infection. C65 pediatric case reports: bronchiectasis, cystic fibrosis, and lung infections: American Thoracic Society; 2017. p. A6106-A.

67. Rozenbaum R, Sampaio MG, Batista GS, Garibaldi AM, Terra GM, Souza MJ, et al. The first report in Brazil of severe infection caused by community-acquired methicillin-resistant *Staphylococcus aureus* (CA-MRSA). Braz. J. Med. Biol. Res. 2009;42(8):756-60.

68. Saglani S, Harris KA, Wallis C, Hartley JC. Empyema: the use of broad range 16S rDNA PCR for pathogen detection. Arch. Dis. Child. 2005;90(1):70-3.

69. Samra T, Yadav A, Banerjee N, Pawar M, Deepak D. Flucytosine for treatment of *Candida albicans* in H1N1-positive patient. Indian J. Pharmacol. 2010;42(5):318-9.

70. Sancho-Tello S, Bravo D, Borrás R, Costa E, Muñoz-Cobo B, Navarro D. Performance of the lightCycler septiFast test M grade in detecting microbial pathogens in purulent fluids. J. Clin. Microbiol. 2011;49(8):2988-91.

71. Sárvári KP, Sántha D, Kovács R, Körmöndi S, Pető Z, Vereb T, et al. Six cases of *Solobacterium moorei* isolated alone or in mixed culture in Hungary and comparison with previously published cases. Anaerobe. 2020;65.

72. Shah SS, Florin TA, Ambroggio L. Procalcitonin in childhood pneumonia. J. Pediatr. Infect. Dis. Soc. 2018;7(1):54-5.

73. Skouras V, Polychronopoulos V, Light RW. Bacteriology of pleural infection. "*Streptococcus milleri* group" in the limelight. Pneumon. 2009;22(1):54-64.

74. Sohn KM. Diagnostic value of synovial fluid adenosine deaminase level in tuberculous arthritis. J. Pak. Med. Assoc. 2021;71(4):1246-8.

75. Sparo MD, Castro MS, Andino PJ, Lavigne MV, Ceriani C, Gutiérrez GL, et al. Partial characterization of enterocin MR99 from a corn silage isolate of *Enterococcus faecalis*. J. Appl. Microbiol. 2006;100(1):123-34.

76. Tchatchouang S, Nzouankeu A, Kenmoe S, Ngando L, Penlap V, Fonkoua MC, et al. Bacterial aetiologies of lower respiratory tract infections among adults in Yaoundé, Cameroon. Biomed. Res. Int. 2019;2019:4834396.

77. Utine GE, Pinar A, Özçelik U, Şener B, Yalçin E, Doǧru D, et al. Pleural fluid PCR method for detection of *Staphylococcus aureus*, *Streptococcus pneumoniae* and *Haemophilus influenzae* in pediatric parapneumonic effusions. Respiration. 2008;75(4):437-42.

78. Vu-Thien H. Empyema: Bacterial epidemiology and antibiotic resistance. Arch. Pediatr. 2008;15(SUPPL. 2):S81-S3.

79. Wiersinga WJ, Bonten MJ, Boersma WG, Jonkers RE, Aleva RM, Kullberg BJ, et al. SWAB/NVALT (dutch working party on antibiotic policy and dutch association of chest physicians) guidelines on the management of community-acquired pneumonia in adults. Neth. J. Med. 2012;70(2):90-101.

80. Wrightson JM, Wray J, Rahman NM, Crook DW. Low Prevalence Of" Atypical" Pathogens In Pleural Infection. D14 Advances in pleural infection and malignancy: American Thoracic Society; 2012. p. A5342-A.

81. Xirogianni A, Tzanakaki G, Karagianni E, Markoulatos P, Kourea-Kremastinou J. Development of a single-tube polymerase chain reaction assay for the simultaneous detection of *Haemophilus influenzae*, *Pseudomonas aeruginosa*, *Staphylococcus aureus*, and *Streptococcus* spp. directly in clinical samples. Diagn. Microbiol. Infect. Dis. 2009;63(2):121-6.

82. Zheng X, O'Leary A, Uhl JR, Patel R, Shulman ST. Rapid detection of *Streptococcus pyogenes* in pleural fluid samples from pediatric patients with empyema. J. Clin. Microbiol. 2012;50(8):2786-7.

83. Zhu RY, Zhang KX, Zhao MQ, Liu YH, Xu YY, Ju CM, et al. Use of visual loop-mediated isotheral amplification of rimM sequence for rapid detection of *Mycobacterium tuberculosis* and *Mycobacterium bovis*. J. Microbiol. Methods. 2009;78(3):339-43.
